# Beyond Missingness: Systematizing Methods for Comprehensive Data Fitness Assessment in Clinical Research

**DOI:** 10.2196/76398

**Published:** 2026-04-14

**Authors:** Hanieh Razzaghi, Kaleigh Wieand, Kimberley L Dickinson, Michael G Kahn, Jason Roy, Clair Blacketer, Dimitri A Christakis, Christopher B Forrest, Jane Greenberg, Harold P Lehmann, Keith A Marsolo, Jennifer Sciolla, Mark G Weiner, Nicole G Weiskopf, L Charles Bailey

**Affiliations:** 1 Applied Clinical Research Center, Children's Hospital of Philadelphia, 3401 Civic Center Blvd, Philadelphia, PA, 19104, United States, 1 814-441-9659; 2 Analytics Resource Center, University of Colorado Anschutz Medical Campus, Aurora, CO, United States; 3 Department of Biostatistics and Epidemiology, Rutgers School of Public Health, Piscataway, NJ, United States; 4 Epidemiology Analytics, Janssen Research and Development, Titusville, NJ, United States; 5 Department of Pediatrics, Seattle Children's Hospital, Seattle, WA, United States; 6 Department of Information Science, Drexel University, Philadelphia, PA, United States; 7 Department of Medicine, Johns Hopkins School of Medicine, Baltimore, MD, United States; 8 Department of Population Health Sciences, Duke University School of Medicine, Durham, NC, United States; 9 Biomedical Research Informatics Center, Nemours/Alfred I. DuPont Hospital for Children, Wilmington, DE, United States; 10 Department of Population Health Sciences, Weill Cornell Medicine, New York, NY, United States; 11 Department of Medical Informatics and Clinical Epidemiology, Oregon Health and Science University, Portland, OR, United States

**Keywords:** data quality, electronic health records, research readiness, automation, data fitness

## Abstract

Secondary use of clinical data offers unprecedented opportunities to rapidly conduct large-scale research and improve patient care. However, incomplete understanding of data quality requirements for a study often causes significant delays in executing analyses and validating results. Current practice has largely followed 2 paths. First, multi-institutional networks have developed general data quality programs, but these are typically tied to unique network characteristics and do not address study-specific requirements well. Second, models have been proposed to formalize the requirements for data fitness analyses without extending to the methods needed to meet these requirements. More recently, tools have been developed to conduct cohort-centric screening, focusing on generally applicable structural checks such as missingness or facial implausibility. These provide a first level of information but incompletely capture the fitness requirements of an analysis. In turn, investigators conduct per-study exploratory analyses, but these efforts are typically ad hoc and partially reported, which can hinder reproducible science and delay advances in patient care. Analogously to advances over the past decade in data modeling and reproducible analytics, there is a need for a more systematic, capable approach to study-specific data quality assessment (SSDQA). We discuss such a model, which guides improved SSDQA design and implementation, including metadata for consistent annotation and reporting of data quality assessment results. The model integrates theoretical principles of data quality testing with pragmatic considerations of application to clinical data, providing a consistent approach to specifying data quality assessment checks. Additionally, it proposes to regularize check application through a standard set of options. The SSDQA model builds on current practice, providing a path toward more complete, sound, and reproducible assessments. These characteristics foster multidisciplinary collaboration to identify data quality issues that, in turn, inform decisions about study design and provide important context that has a bearing on adoption of results.

## Introduction

### Network Data Quality Assessment

Secondary use of clinical data offers unprecedented opportunities to conduct large-scale research and derive insights into patient care. This is particularly true when using data from multi-institutional networks such as PEDSnet [1], PCORnet [2], the National COVID Cohort Collaborative [3], All of Us [4], or others [5-9]. However, clinical data are complex, and problems related to data quality [10-16] have significantly hindered research across many networks. General data quality assessment (DQA) programs have been developed for identifying and resolving issues in real-world data [17-32], including in multi-institutional networks, but efforts have often been siloed by unique network characteristics, differences in data models, or lack of automation and reproducibility [33-35]. This tight coupling of DQA programs with existing infrastructure limits reuse in the wider community.

Data quality frameworks have traditionally focused on data model conformance, completeness, and plausibility components [27] rather than targeting specific clinical content, which can miss significant threats to analytic validity. This is not simply a lack of effective implementation. Rather, it reflects that general DQA at the network level addresses separate analytic problems from study-specific DQA (SSDQA), although the 2 domains are related. For example, a network data quality metric may evaluate clinical procedures as a single group to ensure appropriate mappings and compute procedure records per patient. However, if a particular study relies on the presence of specific biopsy procedures, this can only provide a starting point. A gap in data extraction that maps these procedures to a valid but less specific code or misses these mappings entirely—whether overall or for the study group specifically—would not be discovered during broader network data quality checks. Further testing would be needed to assess whether the needed information could be recovered by changing extraction of procedure data or the presence of the biopsy must be inferred from other data. As a result, most network-focused data quality programs lack the flexibility needed in study-specific contexts. Furthermore, most current DQA research focuses primarily on harmonization of terms to *describe* data quality rather than developing standardized models to *produce* standardized assessments.

### Addressing Study-Specific Data Quality

While less widely considered, there have been several attempts to address the need for domain-specific or SSDQA. Efforts focused on regulatory science targeting the US Food and Drug Administration and European Medicines Agency have produced planning guides for evaluating fitness in real-world data, such as SPIFD (Structured Process to Identify Fit-for-Purpose Data) and STaRT-RWE (Structured Template for Planning and Reporting on the Implementation of Real-World Evidence Studies), which have considerably advanced the field [36-38]. Similar work derived from model-based software design has addressed analogous steps [39]. These frameworks formalize study design and data requirements, underlining what is needed to produce reliable results for regulatory decision-making. However, providing specific methods or software to standardize and systematically report on findings was beyond the scope of these efforts. Furthermore, these and similar frameworks recognize the importance of well-delineated reporting on data quality but focus on reporting as part of a bespoke study design rather than building reusable informatics frameworks or tools.

Separately, several tools have taken pragmatic approaches to interrogating study datasets. Some address specific topics, such as missingness or “never events” in DQe-c [40] or differences in case mix in the ENACT Data Quality Explorer [35]. Another effort, dataquieR [41,42], focuses on highly flexible testing of a priori constraints; the resulting tools have a wide potential range but concretely address a smaller set of data quality needs and provide limited options to probe semantic issues that may arise in the data. A third direction, exemplified by the Observational Health Data Sciences and Informatics CohortDiagnostics package [43], has been to transfer tests that are effective at the network level for application to study cohorts to produce more specific reporting.

While each of these efforts has been a step forward, evaluations of the current state of SSDQA for clinical data have consistently identified the need for more standard, applicable, automatable approaches [33,34]. A recent systematic review of DQAs and tools concluded that most SSDQAs are developed on a project-by-project basis and that this approach is not practical because of time and resource constraints [33]. Finally, prior efforts have focused on solving a specific problem rather than developing an underlying model to serve a wide range of data quality requirements in a systematic and reproducible way.

### Systematizing SSDQA

The challenge, then, is that SSDQA *content* must be specific to the semantic needs of each analysis, whereas SSDQA *methods* need to be standardized and reproducible. The field has advanced significantly in the past decade, but current practice does not fully attain either of these goals. There remains a need to bridge gaps between broad network DQA, high-level proposals for SSDQA needs, and current ad hoc practice of SSDQA. In this paper, we discuss how this challenge can be met, building from existing science including our own previous work [44] and expert consensus to articulate a model for a systematic approach to SSDQA and reporting. Specifically, we focus on the critical space between formalizing the high levels of SSDQA [36-39] and the specifics of implementing tests [35,40,41], aiming to articulate methods that satisfy the former’s needs and systematize approaches to the latter. We address (1) well-founded, analysis-aware data quality testing and (2) the construction of practical, reusable data quality metrics that can be widely adopted to drive consistent and concrete data quality check development. Advancing in these areas is important to clinical informaticians as methodologists and stewards of data resources; to clinical researchers, who are reliant on large datasets to produce valid results; and to patients and clinicians, who must be informed consumers of research results.

## An Expanded Model for Assessing Data Fitness

### Articulating Requirements for Clinical Data Fitness

We first articulate a set of requirements for advancing the practice of SSDQA. We include both high-level strategies, or goals that an effective model must reflect, as well as specific design principles that motivate the process of check construction and application, which are shown in Textbox 1.

Textbox 1. **Design principles for developing study-specific data quality assessment.**

- Data quality assessment should correspond to the different stages of a research project, ranging from cohort selection to assessment of minor covariates.

- The principles of a framework should be standardized and reusable across multiple study-specific contexts, and software packages should streamline implementation.

- Data quality tests need descriptive terms that are specific and pair methods with output.

- Data quality software packages should be configurable without the need for extensive coding.

- Methods used in data quality checks should be interpretable, with a range of standard options to evaluate results.

- Data quality checks should be pragmatic and informative, without requiring deep knowledge of underlying theory for use.

- Temporality should be an important dimension for data quality checks and not be treated as a separate check itself.

**Figure 1.**
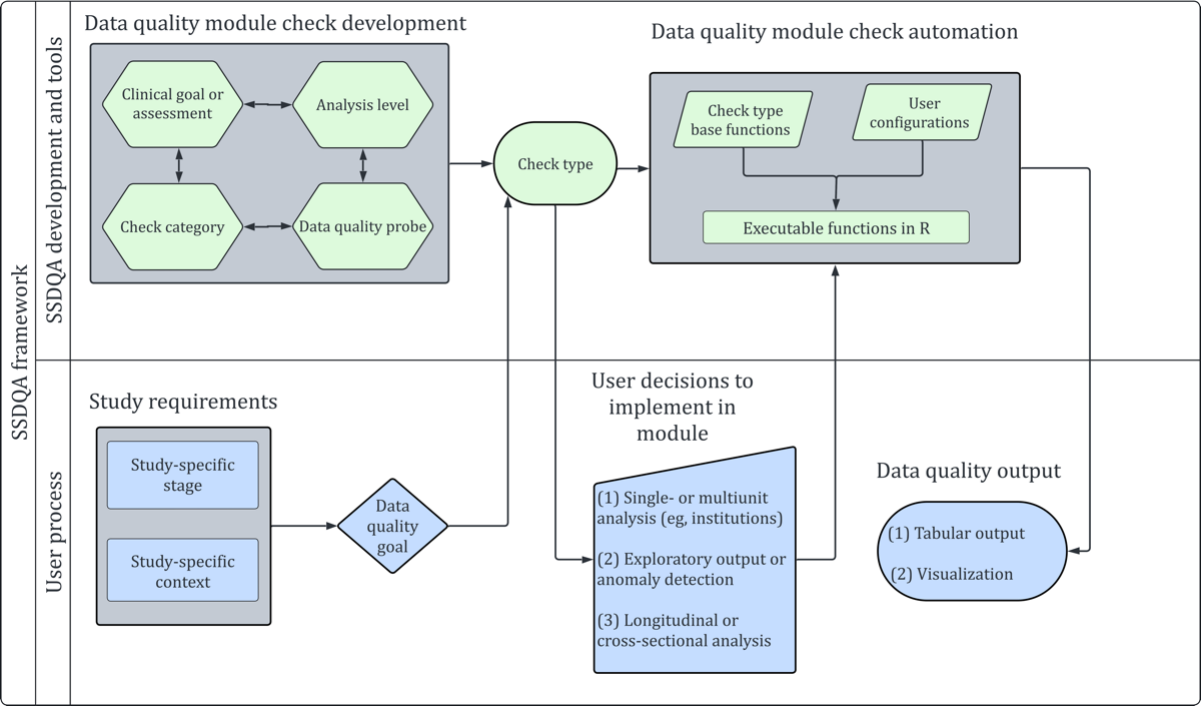
Model for systematic construction of study-specific data quality assessment (SSDQA) checks showing the conceptual tracks of check development and user process and the stages of initial specification followed by application.

We then synthesize these considerations into a concrete model (Figure 1) for constructing fitness-oriented data quality checks. The model provides consistent guidance for standardizing check types that evaluate data fitness across a range of study-specific requirements. It delineates the processes of check development and execution through two interdependent processes labeled on the left-hand side of Figure 1: (1) SSDQA development and tools, which focuses on check development and back-end automation; and (2) user process, which highlights the interaction between the user and data quality checks. We elaborate on these processes in the 2 sections below.

### Adding Rigor to SSDQA Development

To promote better coverage of SSDQA needs as well as improved understanding of what the measures produce, the development of a data quality check type should incorporate 4 separate but integrated components. The first is the clinical goal or assessment, which describes the clinical truth that the user needs to evaluate; this aligns in use with purposive frameworks such as SPIFD [37]. For example, an investigator might posit that patients in a study cohort should have confirmatory clinical data (eg, end-stage renal disease and evidence of dialysis) to establish basic face validity. While check types are broader than specific study goals, what goals a check type could address is an important consideration. The second component is check category, which expresses the evaluated aspect of data quality; here we encompass current work focused on widely adopted harmonized terms [45] such as completeness, plausibility, or conformance. The third component is a data quality probe, which, in contrast to the clinical goal, designates a data-centric purpose for a data quality check, such as assessing eligibility criteria or mapping errors in the data. Finally, analysis level describes the application of a check across levels of aggregation, including person-level, event-level, or visit-level analysis. The integration of these 4 components forms a check type, which is discoverable through these attributes; a set of metadata terms reflecting current practice is provided in Textbox 2. Use of these and similar terms allows check types to be findable for reuse in different studies and promotes interoperability and reusability in the comparison of results across studies.

Textbox 2. **Metadata terms associated with attributes of study-specific data quality assessment check types.**

**Check category**

- Concordance
- Conformance
- Completeness
- Consistency
- Information representation
- Plausibility

**Data quality probe**

- Misclassification
- Eligibility criteria
- Missing expected data
- Anomalous information density
- Temporal inconsistency
- Anomalous value from internal distribution
- Data representation errors
- External benchmarking
- Selection error or bias

**Clinical goal or assessment**

- Clinical consistency
- Clinical follow-up
- Clinical complexity
- Confirmatory clinical data
- Expected clinical event representation
- Targeted patient population
- Utilization patterns
- Valid diagnostic criteria

**Analysis level**

- Person level
- Visit level
- Event level

Once a check type has been specified, application to the data could be mediated by a common code base instantiating the desired analyses, spanning the set of user configurations (see below) required to execute a specific data quality check of that type. This approach, which we term “data quality modules,” facilitates consistent application of the check type through a set of base functions incorporating user choices via parameters without requiring reimplementation or code editing and the consequent risk of divergent computation across use cases [46].

### Standardizing Application of DQA Checks

Application of study-specific data quality testing must by definition be adapted to each study’s requirements. Data quality checks are deployed to inform decision-making about study design and method choices, assess the validity of the dataset, and identify potential biases or stratifications present in the data. Data quality requirements may vary based on the stage of the study or the background of the user. For example, data quality for a clinical researcher planning cohort selection will require a different set of evaluation measures from those for the validation of primary outcomes, and testing at the design stage will differ from a statistician investigating an anomalous result during analysis. This is denoted as “study-specific stage” in Figure 1.

In parallel, “study-specific context” compels users to formally define the data constructs (eg, cohort, variables, and concept sets) around which to identify potential gaps in data quality or constraints in study design that require testing. This step drives the creation of the “study-specific” requirements at the core of SSDQA application. On the basis of these criteria, users go on to select the appropriate check types to be applied to the desired data elements.

Three final decision points determine how a particular check is applied and interpreted. First, applications may focus on single- or multi-unit analysis. Most often, units are participating institutions, and multiunit output facilitates comparisons among institutions, whereas single-unit output permits more in-depth analysis within a particular institution. In other circumstances, it may be more correct to combine all institutions as a single unit of analysis or stratify by other characteristics, such as clinical specialty. Second, a particular data quality analysis may focus on manual exploration or be used to formally detect outliers. The former produces descriptive visualizations, which are particularly useful for assessing patterns relying on topical expertise. In contrast, the latter computes quantitative metrics to identify potential outliers, which is useful when assessing complex patterns or for automation. Third, users may wish to analyze their data longitudinally to understand changes over time or cross-sectionally covering a specified period to examine average effect.

### Augmenting the Scope of SSDQA

This model for SSDQA allows us to consider current practice centered on analytic requirements rather than software output and identify areas for expansion. We begin with a set of 18 data quality check types (Table 1) that cover foundational requirements for SSDQA; while not exhaustive, these check types span a large fraction of use cases encountered in clinical studies. Although many check types can be applied at multiple phases of a study, we group them here based on likely associations. For example, during the “cohort identification” phase of a study, research teams might deploy the “attrition step” or “sensitivity to selection criteria” check types. The primary difference between “cohort fitness” and “dataset fitness” is that the former evaluates the fitness of a specific cohort against study requirements, whereas the latter tests for the logical cohesion of a dataset. Therefore, anomalies found in the former require investigating study-specific criteria, whereas those found in the latter identify broader logical inconsistencies in a dataset. “Data conformance” check types evaluate a dataset for conformance to a required set of standards, such as ensuring that units for a particular laboratory test conform to the standard or accepted reporting for that test. “Variable testing” focuses on study variables, that is, a set of operational clinical definitions represented in an analysis. Nearly all check types can be reasonably configured for single-site vs multi-site analysis, exploratory output vs anomaly detection, and longitudinal vs cross-sectional analysis. As a result, a single check type may yield 8 different types of outputs.

**Table 1.** Catalog of check types derived from the study-specific data quality assessment model.

Check type	Check description	Metadata terms
Cohort identification		
Attrition step	Assess heterogeneity in cohort selection through attrition steps	“Plausibility,” “eligibility criteria,” “missing expected data,” “selection error or bias,” “target patient population,” and “person level”
Sensitivity to selection criteria	Evaluates the impact of alternate cohort definitions by comparing demographics, follow-up time, utilization patterns, outcomes, and other key variables	“Consistency,” “eligibility criteria,” “selection error or bias detection,” “clinical consistency,” “target patient population,” and “person level”
Cohort fitness		
Patient facts	Assesses the availability of clinical events such as drug utilization or laboratory events per year of follow-up	“Completeness,” “missing expected data,” “anomalous information density,” “clinical follow-up,” “utilization patterns,” and “visit level”
Patient event sequencing	Evaluates the plausibility of dates in relation to clinical events (eg, chronic kidney disease diagnosis precedes order for dialysis)	“Consistency,” “plausibility,” “misclassification,” “expected clinical event representation,” “confirmatory clinical data,” “utilization patterns,” and “person level”
Patient record consistency	Tests whether the clinical data in a patient’s record are consistent and confirmatory (eg, patients with leukemia should be receiving chemotherapy)	“Consistency,” “plausibility,” “missing expected data,” “clinical consistency,” “confirmatory clinical data,” “utilization patterns,” “valid diagnostic criteria,” and “person level”
Expected facts present	Evaluates whether a cohort has specific types of clinical data, such as BMI, office visit procedures, or vital signs	“Completeness,” “missing expected data,” “valid diagnostic criteria,” “expected clinical event representation,” and “visit level”
Clinical data values and ranges	Assesses whether clinical data outcomes or values align with patient cohort characteristics	“Consistency,” “misclassification,” “confirmatory clinical data,” “valid diagnostic criteria,” and “person level”
Dataset fitness		
Clinical events and specialty agreement	Evaluates the concordance of specific events (eg, diagnosis of type 1 diabetes) and specialist clinicians or care sites (eg, endocrinologist or endocrinology clinic) at the visit level	“Concordance,” “data representation errors,” “confirmatory clinical data,” “utilization patterns,” and “visit level”
Visit clinical data agreement	Determines whether expected clinical events occur within the same clinical encounter (eg, ventilator support in the ICU^a^ or initial antihypertensive prescription and blood pressure reading)	“Concordance,” “missing expected data,” “clinical consistency,” “expected clinical event representation,” and “visit level”
Date sequencing	Detects outliers and anomalous values such as dates too far in the past or future and whether dates assigned to clinical events occur in a reasonable order or proximity	“Plausibility,” “temporality inconsistency,” “clinical consistency,” “utilization patterns,” and “event level”
Data conformance		
Clinical metadata	Assesses whether clinical facts are accompanied by appropriate metadata (eg, prescription drugs with dosing information and laboratory values with assigned specimens)	“Conformance,” “missing expected data,” “data representation errors,” “expected clinical event representation,” and “event level”
Unit and value alignment	Determines whether drug prescriptions and administrations or laboratory results contain units that are conformant to expected standards	“Conformance,” “misclassification,” “clinical consistency,” and “event level”
Duplicate record check	Identifies where there are duplicate rows or values in a given dataset	“Conformance,” “anomalous information density,” “utilization patterns,” and “event level”
Variable testing		
Expected variables present	Checks for the presence of expected variables and presents a variety of distributions of these variables in the dataset	“Completeness,” “missing expected data,” “external benchmarking,” “expected clinical event representation,” “valid diagnostic criteria,” and “person level”
Quantitative variable distributions	Evaluates quantitative distributions of clinical values (eg, laboratory values or BMI) or patient characteristics (eg, number of visits per patient or follow-up time)	“Plausibility,” “anomalous value from internal distribution,” “valid diagnostic criteria,” “clinical consistency,” and “event level”
Concept set testing		
Concept set distribution	Examines distributions of concepts (eg, codes) that represent a particular variable (eg, wheezing or uncomplicated asthma are the most common concepts represented in primary care cohort, but severe asthma may be the most common in patients with prolonged respiratory specialty care)	“Information representation,” “data representation errors,” “expected clinical event representation,” and “event level”
Source and concept vocabularies	Provides source-to-concept mappings and their distributions to identify potential problems related to information loss	“Information representation,” “data representation errors,” “clinical consistency,” and “event level”
Unmapped concepts	Shows the potential impact of unmapped concepts within a dataset	“Information representation,” “misclassification,” “missing expected data,” “expected clinical event representation,” and “event level”

a ICU: intensive care unit.

Using this taxonomy, we see that current DQA tools operate largely within a few types. Tests for missingness or data density, a focus of many packages [26,27,40,41,43], fall within the “expected facts present” type or, in some cases, assess “clinical metadata” [27]. Tests for never-valid implausibility [26,27,41,43] most often assess date sequencing or, in some cases, patient record consistency. Conformance-oriented packages [17,27,43] may add to these tests matching the “unmapped concepts” and less often the “unit and value alignment” or “duplicate record” check types. The ENACT Data Quality Explorer focuses on diagnosis code distribution [35], a form of the “expected facts present” type, as an opportunity to infer other data quality gaps.

Viewed through this lens, it becomes clear that there is good coverage of structural and study-autonomous aspects of data quality that can disrupt analyses, but there are few resources for systematic interrogation of the clinical semantics that are equally critical to study validity. It is in these areas that we believe that the calls for increased standardization in data quality testing are most apt [33,34].

### Applying Well-Formed DQA to Clinical Research

#### Identifying Requirements During Study Design

To illustrate how the approach that we discuss can improve clinical studies, we examine a specific need in clinical research design: assessing whether persons and data available for a study correctly identify the at-risk population, that is, form a clinically reasonable basis to ask the research question. There are several implicit aspects of this question, including the following: (1) Does the cohort accurately reflect the medical state of interest? (2) Are there undiscovered selection biases or stratifications in the cohort? (3) Are the data elements needed to find patients present? For this discussion, we will focus on the specific question of whether the cohort behaves as one would expect for eligible patients; that is, does it have facial and possibly construct validity? The study development path we propose encapsulates these ideas in a single check type that evaluates whether the *variables* (ie, clinical concepts) that a study’s design expects to find are present in a study dataset (check type: expected variables present), applied during the cohort identification and fitness phases of the study. We note that this is different from the more commonly addressed question of whether *data elements* (ie, labels that ideally capture specific facts for the study) are present; this would follow the “expected facts present” type. The model distinguishes between the 2 because they are semantically different, notwithstanding the fact that they are often confused in practice. Several other check types will be germane to the study’s process of fitness testing. In turn, this should not only inform study design but also produce knowledge that allows others to compare this to other studies and design newer studies more efficiently. However, for the sake of this example, we will limit discussion to 1 check type. We present this as an illustration of how the framework might be used rather than as a systematic evaluation of its effect.

Having identified a relevant check type, we describe the user process phase in a hypothetical study of children with sickle cell disease (SCD). This is an uncommon condition in the general population and, hence, unlikely to drive findings in network-wide data quality testing. However, it is a major cause of both health burden and health care utilization in affected children. For this example, we set 4 expectations: evidence of transcranial Doppler procedure, a standard practice to assess risk of stroke; selected SCD-related laboratory results; pain diagnoses; and hydroxyurea prescription.

#### Single-Unit Analysis

**Figure 2.**
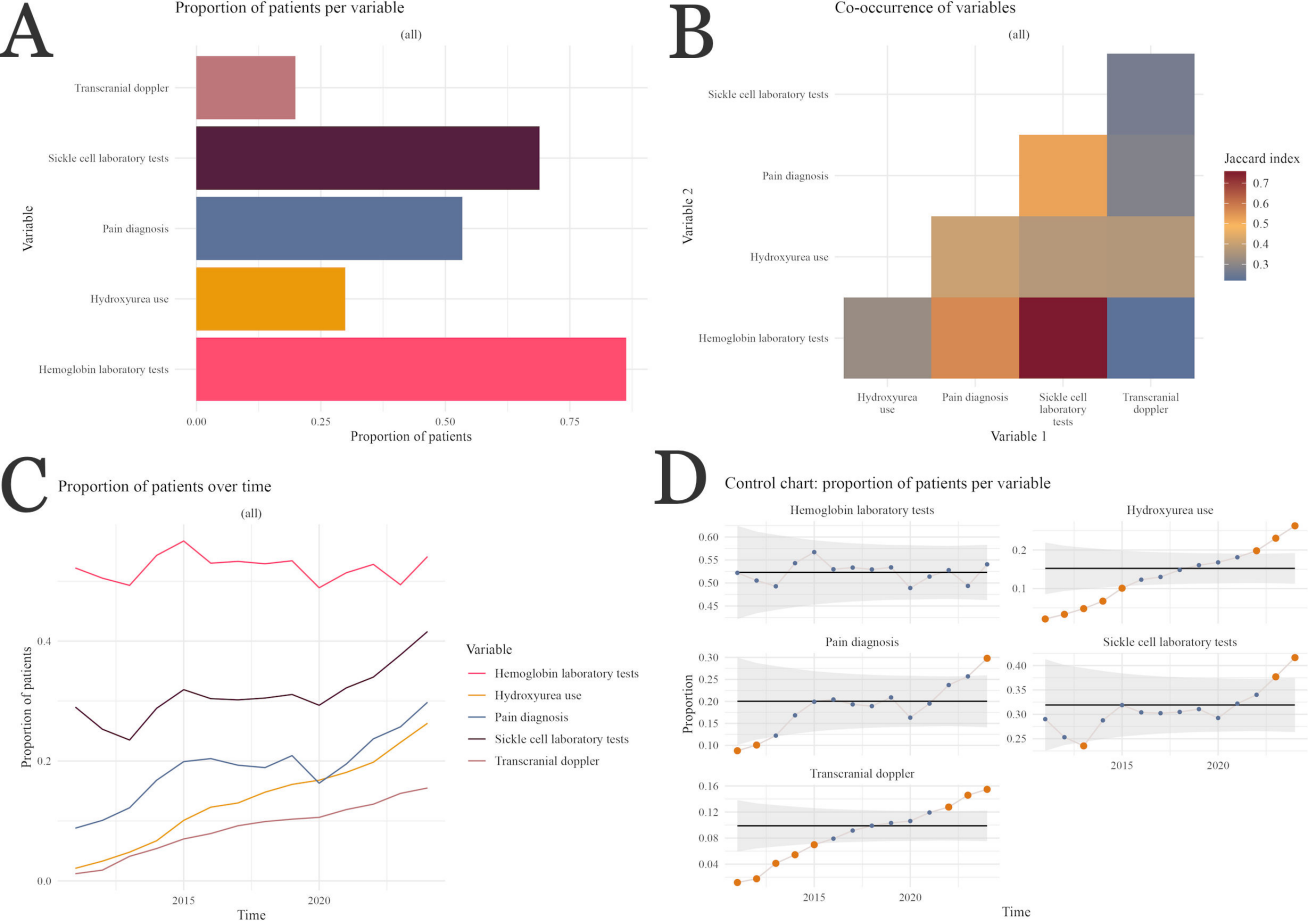
Single-unit results from an “expected values present” study-specific data quality assessment check type: (A) exploratory visualization showing the proportion of the cohort having each of several clinical characteristics that would be expected for the study design, (B) exploratory visualization showing co-occurrence of variable pairs (the Jaccard index is able to account for incomplete capture of each variable, and self-comparisons are omitted), (C) longitudinal trends in completeness of several expected data elements, and (D) anomaly detection using control charts applied to longitudinal trend data.

The single-unit “expected variables present” visualization (Figure 2) allows users to explore data quality as a single unit rather than a comparison of entities to evaluate the dataset in aggregate. Figures 2A and 2B highlight the difference between exploratory visualizations and formal anomaly detection. In this example, distributions of variables indicate that more patients in this cohort have an associated hemoglobin test result (approximately 90%) than other SCD-related tests (approximately 73%). However, there is also a high degree of overlap between patients with hemoglobin and other tests (Figure 2B), and the clinical expectation is that all patients followed for SCD will have results from both sets of tests. This would imply that data quality problems are more common for capturing or representing SCD-specific rather than general test results, as the former are required for a smaller set of patients. Analogously, the use of transcranial Doppler ultrasound, indicated for patients with the more physiologically severe SCD-SS and SCD-Sβ^0^ variants of SCD, correlates fairly well with patients who are taking hydroxyurea, indicated for the same subset of patients, suggesting that patient data are more likely to be complete in this subset of patients requiring more follow-up and clinical observation. The low proportion of patients prescribed hydroxyurea overall may in itself point to a data quality problem, as the drug is indicated for all patients in this subcohort. Possible explanations are that the cohort criteria are too broad (SCD-SS and SCD-Sβ^0^ represent 65%-75% of all patients with SCD) and the drug has only been formally recommended in pediatrics since 2017.

Figures 2C and 2D show temporal trends for the selected variables, which may elucidate whether the low observed proportions for hydroxyurea use are driven exclusively by approval date. Figure 2C is a time-series analysis where all variables are plotted as a proportion of eligible patients over time. It presents relatively stable rates for hemoglobin testing and pain diagnoses, steady increases over time in both transcranial Doppler and hydroxyurea use that reflect updated clinical patient guidelines, and relatively sharp increases in 2013 for laboratory testing variables that may suggest timing of electronic health record implementation rather than differences in care or utilization patterns. Figure 2D shows control charts, with anomalies indicated by orange circles. These graphs reflect similar trends to those exhibited in the exploratory graphs; the hydroxyurea output in particular indicates that the 2017 approval influenced prescribing patterns, although multi-institutional data may clarify whether trends and proportions are heterogeneous. Overall health care utilization still remains lower than expected at approximately 70%.

#### Multi-Unit Analysis

**Figure 3.**
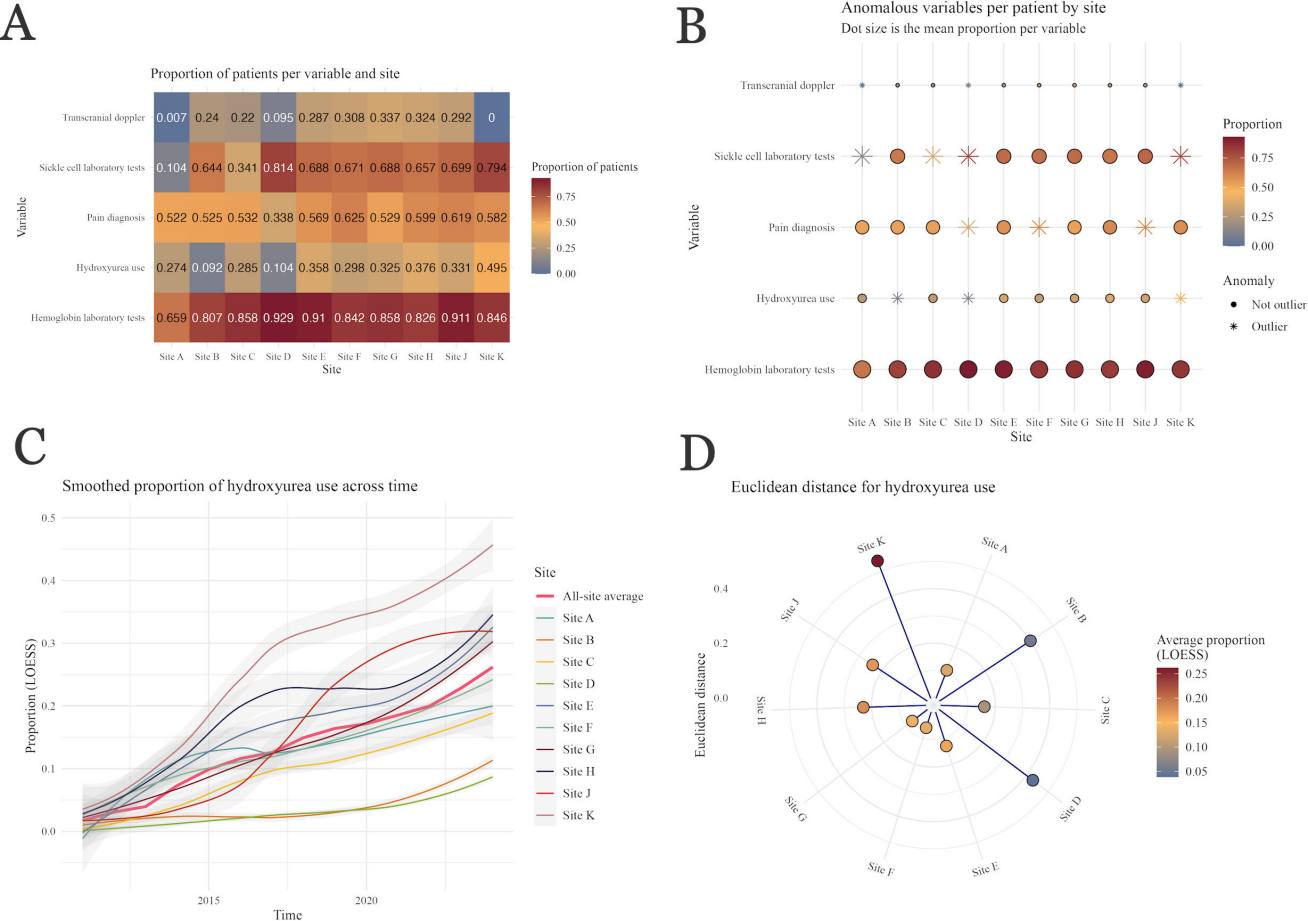
Multiunit results from applying an “expected values present” study-specific data quality assessment check type: (A) exploratory visualization showing the proportion of the cohort having each of several clinical characteristics that would be expected for the study design, now stratified by institution; (B) analogous visualization identifying sites with statistically anomalous proportions for each variable; (C) smoothed (locally estimated scatterplot smoothing [LOESS] with 95% CI) longitudinal trends in the proportion of patients receiving hydroxyurea; and (D) use of the Euclidean distance to summarize the differences in longitudinal trends shown in the prior graph to facilitate identification of outliers.

Figure 3 again shows both the exploratory and anomaly detection visualizations for the same variables in a multiunit (site contributor) analysis. The exploratory visualization shows a heat map with proportions of patients who have evidence of the indicated variable in the dataset (Figure 3A). The display showing tests for anomalous values shows a dot plot with the same color scale but with a star indicating statistical anomalies and the size of the dot indicating the mean proportion across all institutions (Figure 3B). The size and color of the hemoglobin results indicate that all sites capture this variable well, ranging from 78% (site A) to 95% (site J). Site A shows lower-than-expected capture of non–diagnosis-related clinical data, whereas site D captures laboratory tests well for patients but, conversely, captures pain diagnoses, hydroxyurea use, and transcranial Doppler poorly compared with other sites. Notably, site K captures all use well except for transcranial Doppler, likely marking a problem with extracting procedure data from clinical source systems. These graphs illustrate the most frequent anomalies for transcranial Doppler, SCD-specific laboratory tests, and hydroxyurea utilization variables. Hydroxyurea in particular illustrates how heterogeneous data capture or clinical practice might appear, with sites B and D showing low representation (approximately 11%) and sites H and K showing high representation (42% and 52%, respectively).

Finally, we illustrate trends across time, facilitating deeper exploration of the heterogeneity shown in the overall results. Taking hydroxyurea use as an example, Figure 3C reinforces the single-unit observation that the Food and Drug Administration drug approval in 2017 increased use across time. Site J appears the most impacted, with a sharp uptick in 2017 and stable use afterward, in contrast to other sites’ steady rise. The longitudinal data also reinforce the observation that sites B and D have a low proportion of patients prescribed hydroxyurea, and while slightly increasing in more recent years, the proportions remain nearly constant. Site B is shown with high-quality data in other domains, as would be observed with drug-specific data quality problems. In contrast, site D shows anomalous data across domains. Figure 3D displays the overall distance between each site and the all-site median across time. This visualization reveals that sites K, B, and D trend markedly differently from the all-site median, with site K trending higher and, as expected, sites B and D trending lower.

Taken together, this single check type (expected variables present) can produce diagnostic outputs that inform decision-making about several potential data quality problems related to study-specific requirements. Some of these findings would doubtless be identified by a study team performing ad hoc exploratory analyses but likely not all, and certainly with limited potential for cross-study reuse of either tests or results. Importantly, we describe check types designed to augment rather than replace the type of information available from network-wide DQA, allowing for interrogation of semantics critical to proposed analyses and not just data structure.

## Summary

Structuring the ways in which we approach SSDQA contributes to better and more systematic research in several ways. First, checks can be driven by a theoretically sound framework that integrates clinical and technical considerations while retaining the opportunity to configure how it is applied to meet the requirements of the user. Second, the adoption of a common framework enables reproducibility and standardization of SSDQA checks for dissemination in a wider community in keeping with principles of findability, accessibility, interoperability, and reusability [47,48]. Third, the adoption of a shared and well-grounded framework further facilitates transparency about data beyond simple descriptions when sharing of datasets is not possible due to patient privacy considerations. Consumers of research results are better able to understand data characteristics that may have a bearing on the applicability of the results to their circumstances. Similarly, other researchers can account for differences in data source to validate or augment previous work. For example, complex differences in use of the health system across cohorts may bias study outcomes, which is only crudely reported in current publications.

The problem is complex, and efforts will necessarily be incremental for many reasons. First, the universe of potential SSDQA testing is quite large, and while the theoretical model we discuss is expansible, a finite set of check types cannot cover all possible requirements. However, SSDQA for any single study will exercise only a subset of those possibilities; the check types we highlight cover most high-frequency cases. Second, effective SSDQA requires an interdisciplinary team comprising technical, methodological, and clinical domain expertise; systematizing DQA methods does not replace the need for this expertise. Nonetheless, for such teams, a repeatable approach is particularly useful both for study-wide screening for risks and when a DQA issue is suspected. For the former, check types for cohort identification and variable exploration may inform study design, variable definitions, and analytic requirements. For the latter, data anomalies can be investigated during a study in a more predictable way that will not require designing novel checks when time is of the essence. Third, a greater diversity of check types brings with it complexity, requiring familiarization to make the most effective use of available resources. While adoption of well-founded methods is advantageous in the longer term, a culture shift will be needed in areas where practice is currently ad hoc. Finally, our discussion currently focuses on structured data. Given the rapid increase in the use of free-text or imaging data, translation of these ideas will be needed. Nevertheless, the check type components apply across data domains even if the subsequent development of tools differs.

Effective SSDQA is a critical but complex part of conducting valid research using clinical data. As with other steps of the research process, SSDQA benefits from evolving methodological practices. We provide an opportunity to reflect on ways to improve SSDQA effectiveness and reporting. Additional work will undoubtedly be needed as clinical research methods and data sources continue to advance.

## Abbreviations

DQA: data quality assessment
SCD: sickle cell disease
SPIFD: Structured Process to Identify Fit-for-Purpose Data
SSDQA: study-specific data quality assessment
STaRT-RWE: Structured Template for Planning and Reporting on the Implementation of Real-World Evidence Studies
